# Expanding the Monolayer Scope for Nucleic Acid-Based Electrochemical Sensors Beyond Thiols on Gold: Alkylphosphonic Acids on ITO

**DOI:** 10.1149/2754-2726/acc4d9

**Published:** 2023-03-29

**Authors:** Alexander Shaver, Netzahualcóyotl Arroyo-Currás

**Affiliations:** 1 Department of Pharmacology and Molecular Sciences, Johns Hopkins University School of Medicine, Baltimore, Maryland 21202, United States of America; 2 Department of Chemical and Biomolecular Engineering and Institute for NanoBioTechnology, Whiting School of Engineering, Johns Hopkins University, Baltimore, Maryland 21218, United States of America

## Abstract

Electrochemical biosensors are a powerful and rapidly evolving molecular monitoring technology. Evidenced by the success of the continuous glucose monitor in managing Type 1 Diabetes, these sensors are capable of precise, accurate measurements in unprocessed biological environments. Nucleic acid-based electrochemical sensors (NBEs) are a specific type of biosensor that employs the target binding and conformational dynamics of nucleic acids for signal transduction. Currently, the vast majority of NBEs are fabricated via self-assembly of alkylthiols on Au electrodes. However, this architecture is limited in scope, as Au electrodes are not universally deployable for all potential NBE applications. Here, to expand the repertoire of materials on which NBEs can be made, we describe the multistep procedure for creating sensing monolayers of alkylphosphonic acids on a conductive oxide surface. Using such monolayers on indium tin oxide (ITO)-coated glass slides, we couple redox reporter-modified nucleic acids and demonstrate signaling of procaine-binding NBE sensors in buffer and human serum. We investigate the operational stability of these NBE sensors to reveal faster signal loss relative to benchmark thiol-on-gold sensing layers, a result that arises due to poor stability of the underlying ITO. Finally, we discuss future directions to continue expansion of NBE sensor materials and applications.

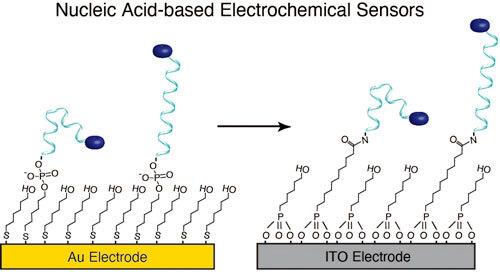

Since the development of the glucose monitor, the field of electrochemical biosensors has seen rapid advancement towards the creation of new molecular monitors. Due to several functional properties including low background in biological fluids,^1^ low cost compared to optical sensors,^2,3^ and ability to be miniaturized,^4^ electrochemical biosensors are ideal for supporting both research and clinical applications. For example, sensors could be interfaced with prosthetics to monitor infection, inflammation, or immune status.^5,6^ They could be incorporated into surgical scaffolds to track healing progression after reconstructive surgery.^7^ They could also be used independently as subdermal implants or wearable devices to track disease-specific therapeutics and biomarkers.^8,9^ Beyond the realm of healthcare, electrochemical biosensors could be embedded in food processing machinery to monitor contamination or purity.^10^ They could also serve as environmental monitors for the presence of toxins or pesticides in crops.^11^ Each of these applications, however, may require different supporting materials on which the sensors are fabricated. Therefore, there is a need for increasing the scope of electrode materials for electrochemical biosensors.

Nucleic acid-based electrochemical (NBE) sensors are a class of biosensor that is particularly suited for continuous molecular measurements in complex environments.^12,13^ They also represent a rapidly-growing field with potential to address global health issues.^14^ NBE sensors rely on both the affinity of synthetic oligonucleotides for specific molecules, and their dynamics upon target binding for signal generation.^15^ In more detail, NBE sensors comprise an electrode surface onto which is deposited a mixed self-assembled monolayer of: I) blocking short-chain alkylthiols to prevent unwanted reactions at the electrode surface, and II) alkylthiol- and redox reporter-modified oligonucleotides (Fig. 1A).^16^ When target is present, the electrode-attached oligos undergo conformational changes that alter electron transfer between the electrode and redox reporter, causing current changes easily measured via electrochemistry (Fig. 1B).^17^ Because this process does not require the addition of exogenous reagents, NBE sensors are ideally designed for continuous measurements.^18^ The approach has already been successfully demonstrated for continuous molecular monitoring in complex environments such as in live rat blood vessels^9,12^ and mice brains.^19^

**Figure 1.**
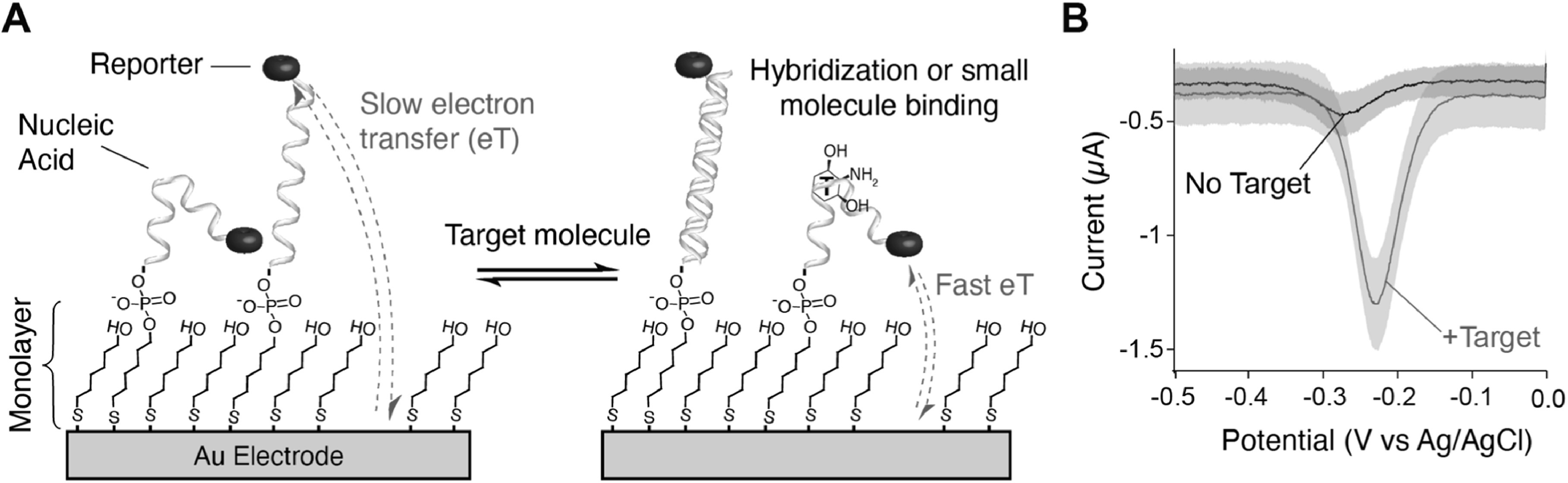
NBE Sensor Anatomy and Signaling. (A) Traditional NBE sensors are fabricated on gold electrodes with a mixed self-assembled monolayer of I) short chain alkylthiols, most commonly 6-mercaptohexanol (shown here), and II) thiolated oligonucleotides modified on the distal end with a redox reporter, most commonly methylene blue. Target binding to oligonucleotides induces a conformational change that alters the distance between redox reporter and electrode surface, thereby altering electron transfer kinetics. (B) When interrogated with square-wave voltammetry, changes in target concentration can be seen as a change in the peak current or area under the voltammogram.

To this day, the majority of NBE sensors have been fabricated on gold electrodes via self-assembly of alkylthiol monolayers.^20^ Unfortunately, gold is not universally deployable in vivo. For example, gold is not transparent to magnetic resonance imaging (MRI), which could impede sensor deployment in conditions where both imaging and molecular monitoring are better used in tandem^21^ (e.g., cancer therapy and traumatic brain injury). Thus, there remains a need to expand the number of materials available to support NBE sensing. In this work, we seek to expand the repertoire of materials on which NBE sensors can be fabricated by evaluating the formation of sensing monolayers on indium-tin oxide (ITO), which is transparent, MRI transparent,^22^ and able to be deposited on polymers, glass, and metallic surfaces.^23,24^ We focus specifically on monolayers of alkylphosphonic acids, as they have been demonstrated to be more stable than thiol on Au monolayers,^25^ and have previously supported the formation of electroactive monolayers.^26^ By harnessing the interactions between alkylphosphonic acids and ITO’s conductive oxide surface,^27^ we created self-assembled monolayers onto which redox reporter-modified oligos can be tethered (Fig. 2A). Additionally, we investigated the operational signaling and stability of these ITO-supported NBE sensors. Although their stability at negative voltages (vs Ag|AgCl) is inferior to that of analogous gold-supported sensors because of the dissolution of indium,^28^ our demonstration of NBE signaling on ITO presents a functional strategy towards the creation of NBE sensors on conductive oxide surfaces.

**Figure 2.**
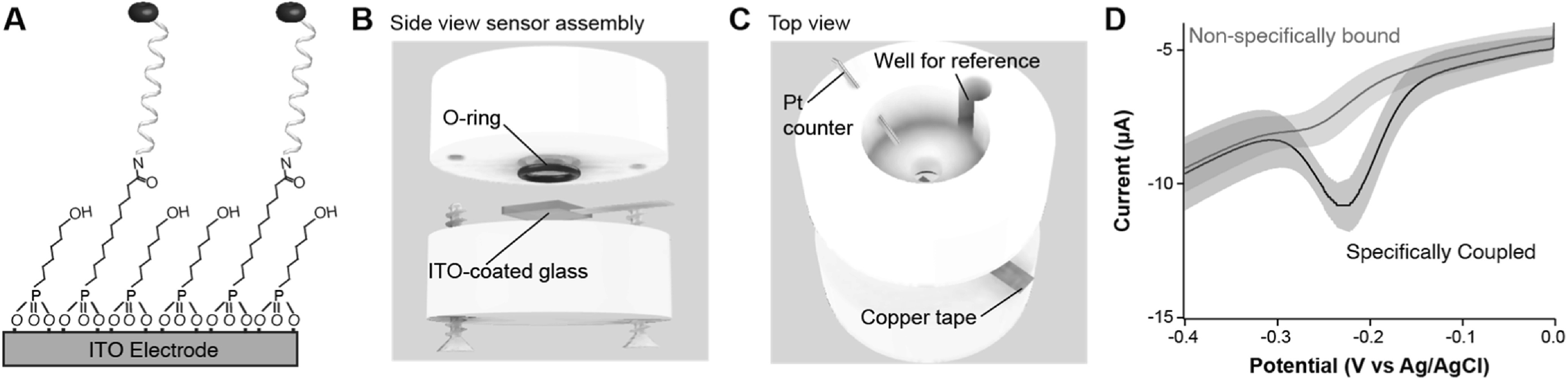
ITO NBE Sensor Assembly and Interrogation. (A) In contrast to the gold supported NBE sensors shown in Fig. 1A, here we fabricated sensors using alkylphosphonic acid monolayers on an ITO-coated glass slide. (B) To support sensor fabrication and testing, we built custom cells sandwiching the modified ITO-coated glass and an O-ring between a top solution holder made of polytetrafluoroethylene (PTFE, also known as Teflon) and a flat acrylic base. (C) The electrode assembly consisted of three electrodes, including a platinum counter, a Ag|AgCl reference, and a the modified ITO substrate as the working electrode. Electrical connection to the ITO was made via a piece of copper conductive tape. (D) Using an alkyklphosphonic acid concentration ratio of 100 *μ*M 11-phosphoundecanoic acid to 1 mM 6-hydroxyhexylphosphonic acid during monolayer formation, our fully fabricated sensors display strong voltammetric signal only for those surfaces exposed to reporter-modified nucleic acids in the presence of coupling reagent. SWV was performed in PBS with a 25 mV amplitude and 60 Hz frequency. Shaded areas represent the standard deviation calculated from 4 independent electrodes.

## Results

### Alkylphosphonic acids enable modification of ITO-coated surfaces

While traditional NBE sensors are fabricated via the self-assembly of short-chain alkythiols and alkythiol-modified nucleic acids onto a gold electrode,^15,16^ either sequentially or simultaneously,^20^ fabrication of sensors on ITO requires a multi-step process to ensure chemical bonding of the phosphonates to the oxide surface without degradation of the nucleic acids. In this work, we developed a five-step fabrication method based on previous reports^29,30^ and our own experimental results. To interrogate electrodes, we employed a custom cell pressing ITO-coated glass and a rubber O-ring between a polytetrafluorethylene (PTFE) top solution holder and a flat acrylic base (Fig. 2B). The cell assembly was held in place via lateral screws. For long-term measurements, cells were also covered with parafilm to prevent solvent evaporation and maintain constant electrolyte concentrations. All measurements reported in this work are based on a three-electrode cell configuration, using a platinum wire counter electrode, a Ag|AgCl reference electrode, and the modified ITO as the working electrode (Fig. 2C). Analysis of fully functionalized surfaces via square wave voltammetry (SWV), a technique that differentially removes capacitive currents, reveals a strong voltammetric redox peak that can be used for sensing applications (Fig. 2D). Unless otherwise noted, all experiments were done in 1x phosphate-buffered saline (PBS). All measurements represent the average and standard deviation between four fully assembled cells, interrogated simultaneously via a multichannel potentiostat. Additional experimental details are provided in the methods section.

**Figure 3.**
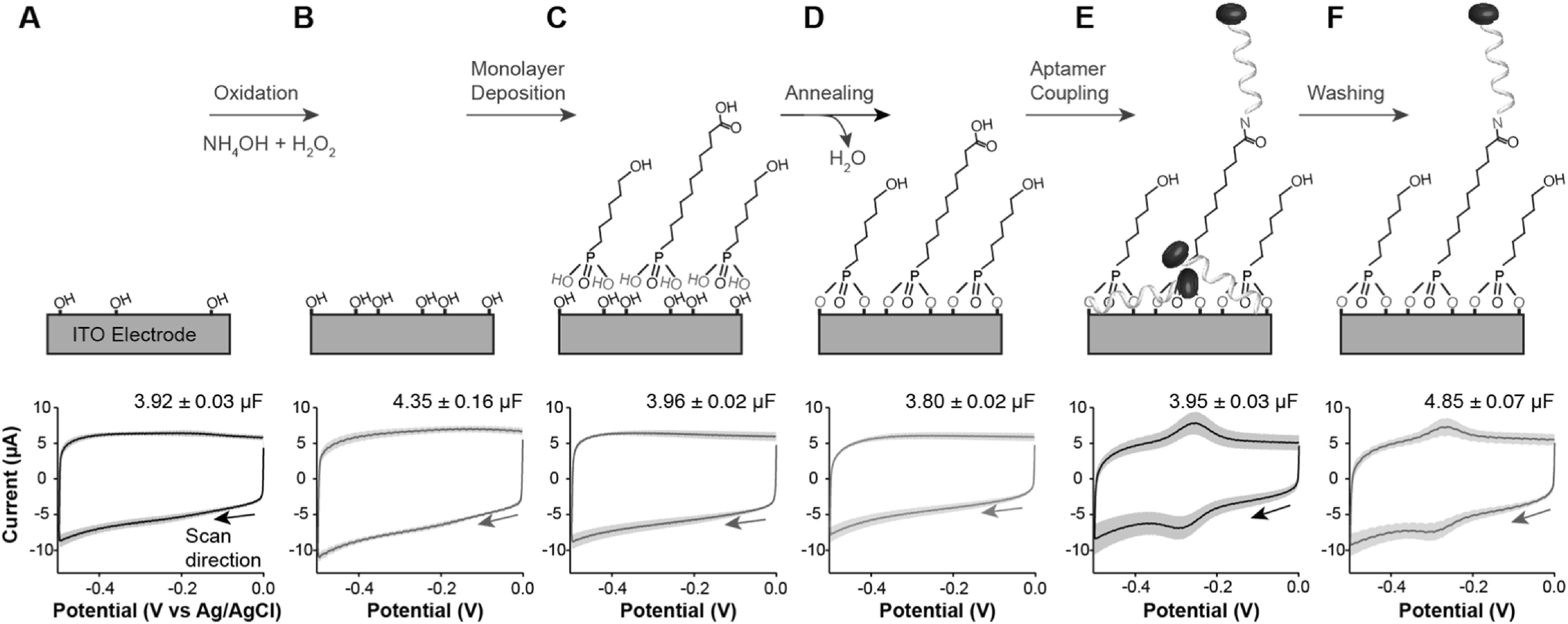
ITO surface modification strategy. Here we present a scheme (top) and cyclic voltammograms (bottom) corresponding to each step in our sensor fabrication procedure. Beginning with commercially available ITO-coated glass slides (A), we oxidize the surface via 20 min sonication in basic piranha (B), incubate overnight in an aqueous solution of 100 *μ*M PUA/ 1 mM HHPA (C), anneal the monolayer for 4 h at 140 °C (D), couple 3’ methylene blue-, 5’ primary amine-modified aptamers (E), and remove non-specifically bound molecules via 30 min sonication first in acetonitrile, then in 6 M guanidine hydrochloride in 80% ethanol (F). The voltammograms were recorded at a scan rate of 1 V/s. The number in the top right corner of each voltammogram is the average surface capacitance of ITO-coated slides at the corresponding fabrication step, as determined in Fig. S1. Shaded areas represent the standard deviation calculated from 4 independent electrodes.

The first step of our sensor fabrication process consisted of oxidizing the surface of commercially acquired ITO-coated glass slides via sonication for 20 min in basic piranha solution (i.e., a 3:1 mixture of NH_4_OH and H_2_O_2_).^29^ This step produces a highly oxidized surface as indicated by the increase in capacitive currents upon interrogation of the electrodes with cyclic voltammetry (CV) (Fig. 3B vs A, Fig. S1B vs A). Much like with impedimetric methods, the capacitive currents in CV (i.e., the hysteresis of the voltammograms) reflect changes to the surface composition of the electrodes. Assuming a model of a parallel plate capacitor, in which the ITO film represents the bottom plate and the electrolyte the top plate, the space between the two plates dictates the magnitude of such capacitive currents (i.e., the diffuse layer is the dielectric).^31,32^ Oxidation of the ITO layer produces hydrated oxide species that reduce the length of the diffuse layer by shielding better the electric field. Because of the inverse relationship between capacitance and distance: C = *k*·*ε*_0_·*A*/*d* (where *k* is the relative permittivity of the medium, *ε*_0_ the permittivity of space, *A* the plate area, and *d* the distance separation between plates), voltammetric capacitive currents increase with decreasing diffuse layer length. In other words, a more hydrophilic surface corresponds to a higher capacitance measurement. Thus, sensor modification steps can be effectively monitored by tracking changes in capacitive currents. To determine the capacitance of our ITO-coated surfaces at each step in the fabrication process, we recorded cyclic voltammograms at various scan rates in the window of 0 to +0.2 V vs Ag|AgCl. Extracting the current at +0.198 V and plotting it against scan rate creates a linear relationship, the slope of which is the surface capacitance (Fig. S1). These values are also presented with the corresponding fabrication steps in Fig. 3.

To form self-assembled monolayers on ITO, the second step is to submerge the glass slides in aqueous solutions of phosphonic acids.^30,33^ In this work, we employed 11-phosphoundecanoic acid (PUA) and 6-hydroxy-hexylphosphonic acid (HHPA) to create a mixed monolayer containing electrode blocking elements (HHPA) and nucleic acid anchoring elements (PUA). After testing four different concentration ratios (500 *μ*M:500 *μ*M, 200 *μ*M:1 mM, 100 *μ*M:1 mM, and 50 *μ*M:1 mM) of PUA:HHPA in the deposition bath, we determined that the 100 *μ*M:1 mM condition achieved the best signal to noise ratio for fully fabricated sensors (Fig. S2A), while maintaining minimal non-specific binding of nucleic acids (Fig. S2B). Following an overnight (∼16 h) incubation in 100 *μ*M:1 mM PUA:HHPA, we observe a measurable decrease in charging current and surface capacitance upon voltammetric interrogation (Figs. 3C, S1C). This observation confirms that a monolayer is formed, increasing the distance between the ITO surface and the electrolyte (i.e., increasing dielectric length), and thereby decreasing capacitive currents.

Unlike thiol self-assembly on gold, chemisorption of alkylphosponic acids requires a dry annealing step to evaporate interfacial water and promote covalent bond formation with the oxide surface.^27^ We perform this third step by incubating the ITO substrates in a dry oven at 140 °C for 4 h. Voltammetric interrogation after this annealing process (Figs. 3D, S1D) further decreases charging current and surface capacitance, an indication that tighter monolayer packing is achieved after high temperature exposure. Next, we functionalize the exposed carboxylic acid groups from the surface-attached PUA via carbodiimide coupling to either new methylene blue (not shown in Fig. 3) or 5’ amine- and 3’ methylene blue-modified nucleic acids. To demonstrate successful amide coupling, we first show covalent bonding of modified oligos to the PUA. For this demonstration, we incubate PUA-coated surfaces in a 1 *μ*M solution of modified nucleic acids overnight (∼16 h) in the presence of 20 mM carbodiimide reagent (EDC). Following this step, we observe voltammetric peaks corresponding to the reversible redox conversion of DNA-bound methylene blue to leucomethylene blue, in addition to an increased surface capacitance indicative of a more hydrophilic surface (Figs. 3E, S1E).

The final step of our sensor fabrication process involves removal of non-specifically bound reporters/nucleic acids from the functionalized surfaces. To remove these molecules, we sonicate the sensor substrates for 30 min each in both acetonitrile and a 6 M solution of guanidine hydrochloride in 80% ethanol. Voltammetric interrogation after these washing steps reveals a decrease of faradaic current coupled with an increase in capacitive current relative to the previous step (Fig. 3F vs E, Fig. S1, F vs E), indicating successful removal of non-specifically bound molecules. Notably, when monolayer-functionalized ITO electrodes are exposed to reporter-modified nucleic acids in the absence of EDC (i.e., a negative control), the subsequent washing steps cause almost complete loss in faradaic current from methylene blue, indicating that non-specifically bound nucleic acids (Fig. 2D) are removed by the washing steps.

### The operational stability of ITO-based sensors is inferior to analogous gold-supported sensors

**Figure 4.**
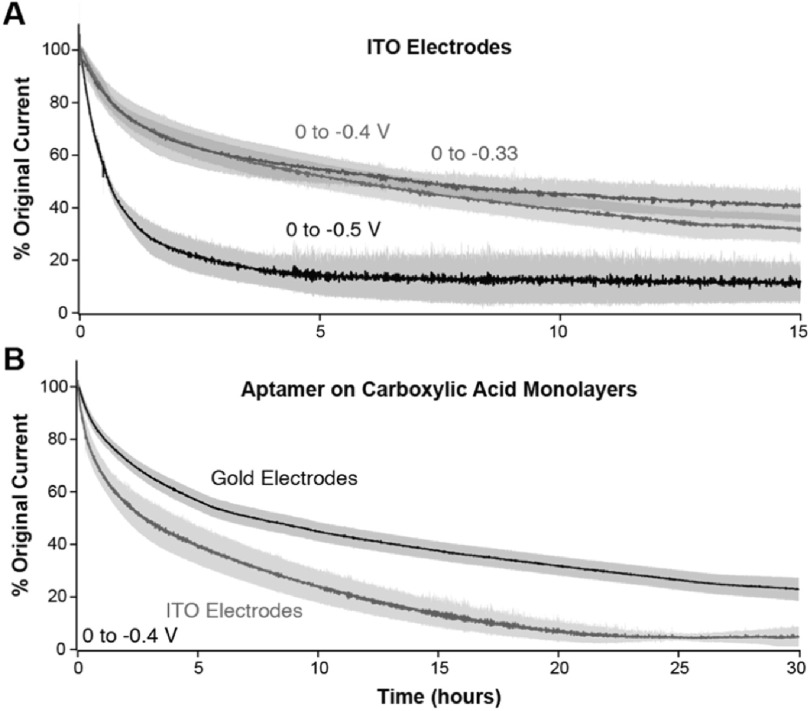
Operational Stability of ITO-supported Sensors. (A) By interrogating ITO-supported sensors every 8–15 s across 3 different potential windows, we demonstrate that minimizing the voltage window used during voltammetric interrogation prolongs their signaling half-life. (B) Using a 0 to −0.4 V vs Ag|AgCl potential window and interrogating every 10 s, we demonstrate here that Au supported sensors decay in signal at a slower rate than ITO supported sensors. All measurements performed by SWV using a 25 mV amplitude and 60 Hz frequency. Shaded areas represent the standard deviation calculated from 4 independent electrodes.

Given that a major limitation of current NBE sensors is their progressive signal loss over time^34^—due in part to electrochemically induced monolayer desorption^35,36^—we first sought to determine the stability of our newly formed alkylphosphonic acid monolayers upon continuous electrochemical interrogation. Interrogating new methylene blue-modified monolayers via SWV (amplitude = 25 mV, frequency = 60 Hz) across three different potential windows, we found that stability is modulated by the potential window applied (Fig. 4A). Specifically, minimizing the potential window to not go beyond −0.4 V vs Ag|AgCl prolonged the operational lifetime of monolayers, a result that has been observed previously with traditional NBE sensor architectures.^37^

Using the window of 0 to −0.4 V vs Ag/AgCl, we then directly compared the signal loss between Au- and ITO-supported sensors. For these experiments, we fabricated analogous sensors on gold electrodes by forming a mixed alkylthiol monolayer from a solution of 100 *μ*M mercaptoundecanoic acid (MUA) and 1 mM 6-mercapto-1-hexanol (MCH), then attached amine- and methylene blue-modified nucleic acids via carbodiimide coupling. Interrogating the sensors by SWV (same technique parameters) every 10 s, we demonstrate that ITO-supported sensors fall below 20% signal within 15 h of continuous interrogation (Fig. 4B). Au-supported sensors, in contrast, retain >20% signal for 30 h, indicating that this more traditional sensor architecture has a longer operational half-life than ITO-supported sensors. However, the short half-life of ITO sensors may not be due to monolayer loss over time. Instead, as discovered by Geiger et al.,^28^ ITO itself is unstable under electrochemical interrogation and leeches into solution when polarized to negative potentials. Additionally, the lability of ITO has been measured at open circuit potential, an indication that these electrode surfaces are not stable even in the absence of voltage biasing. Therefore, we believe that degradation of the ITO, not the alkylphosphonate monolayers, occurs upon voltammetric scanning of the NBE sensors. Of note, the work from Geiger et al. hints towards fluorine-doped tin oxide (FTO) as resisting degradation better than ITO. However, significant efforts yielded an inability to form alkylphosphonate monolayers on FTO-coated glass slides (Fig. S3). Though we are unsure exactly why this is the case, we suspect the increased roughness of FTO may play a part.^35^ For example, one study found that equivalently deposited FTO and ITO had a root mean squared roughness of 16 nm and 0.63 nm, respectively.^38^ It is possible that a higher roughness prevents proper monolayer formation on our FTO-coated glass slides.

### Signaling capability on ITO is sequence dependent

**Figure 5.**
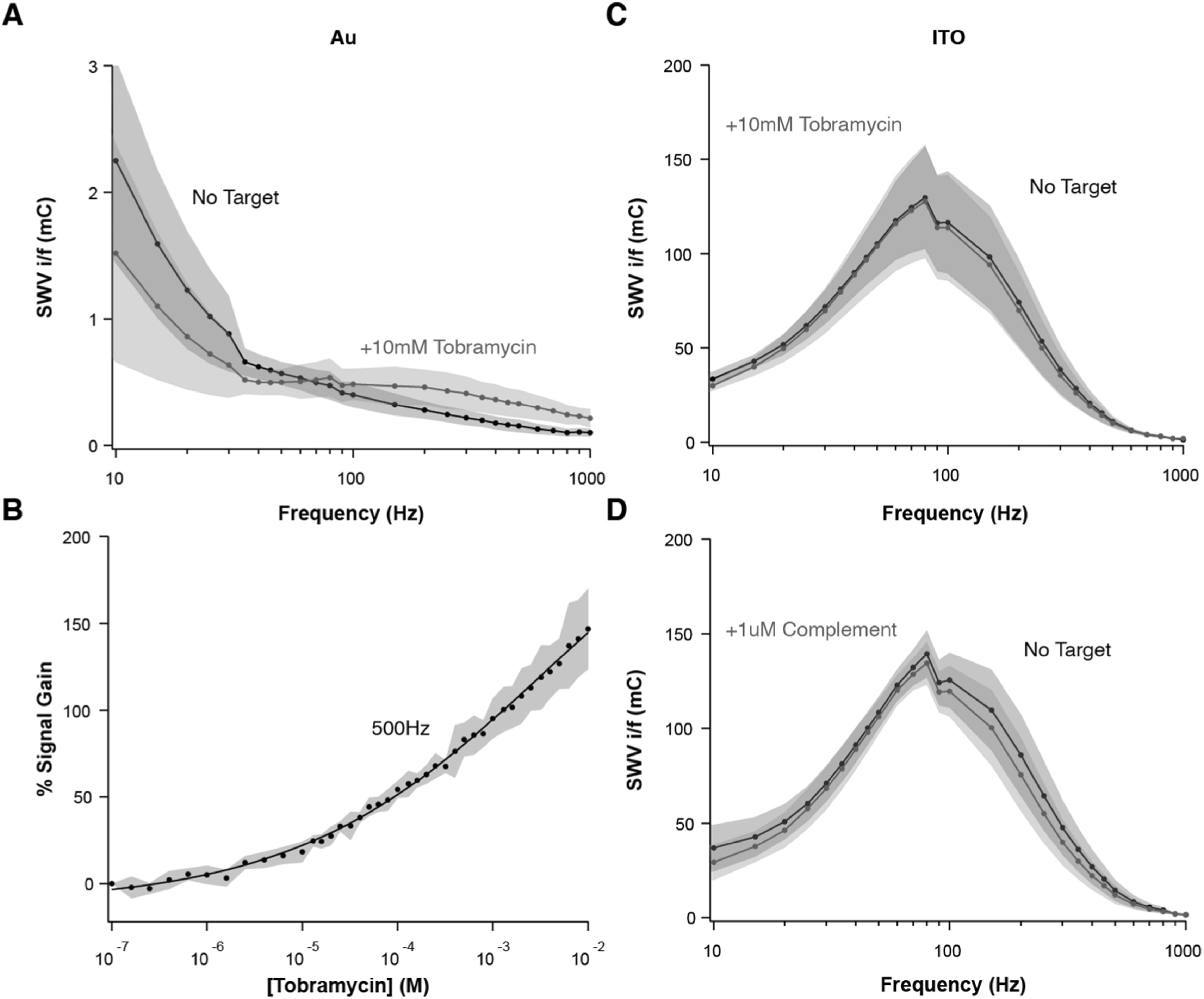
Sensor Signaling with the Aminoglycoside Aptamer. (A) By interrogating aminoglycoside-binding sensors at 27 SWV frequencies, in the absence and presence of a saturating amount (10 mM) of tobramycin, we demonstrate here that Au-supported sensors increase signal upon target binding when interrogated in the range of 200–1000 Hz. (B) Using a frequency of 500 Hz, we then serially interrogate sensors at increasing concentrations of tobramycin to build a calibration curve with ∼150% signal gain and K_D_ = 1.47 ± 1.77 mM. (C) Unlike Au sensors, ITO-supported sensors display no change in signal upon binding to tobramycin, indicating a lack of aptamer dynamics. (D) This lack of signaling remains true upon addition of a single-stranded oligonucleotide complementary to the surface-attached strand. All SWV was performed in the potential window of 0 to −0.4 V vs Ag|AgCl, with an amplitude of 25 mV. Shaded areas represent the standard deviation calculated from at least 4 independent electrodes.

Despite the faster decay of sensor signal on ITO electrodes compared to Au electrodes, there remains value in developing NBE sensors supported by electrode materials other than Au. As a transparent material capable of being deposited on a variety of surfaces, ITO presents a versatile option with which to create sensors for a variety of applications where continuous voltammetric interrogation is not required. With this in mind, we fabricated analogous sensors on ITO and Au via amide coupling of aminoglycoside-binding aptamers (Table 1)^39^ to carboxylic acid groups on alkylphosphonate and alkylthiol monolayers, respectively, as an initial test. To first identify the optimal square-wave frequencies for interrogation of these sensors, we built “quasi-reversible maximum” maps as initially described by Lovriç and colleagues (Figs. 5A, C).^40,41^ Creating these maps in the absence and presence of a saturating amount of tobramycin as target, we can discern a change in the electron transfer kinetics for Au-supported sensors, but not for ITO sensors, upon target binding. Specifically, there is a signal increase for Au sensors binding tobramycin when they are interrogated in the range of 200–1000 Hz (Fig. 5A), which enables the creation of a calibration curve (Fig. 5B). To build this curve, we repetitively interrogated Au-supported, aminoglycoside-binding sensors at 500 Hz, while incrementally increasing the concentration of target tobramycin. For this specific sensor architecture, we reveal a gain of ∼150% and an apparent dissociation constant of 3.47 ± 1.77 mM. These signaling parameters may change at different temperatures, which has been demonstrated before,^42^ but such analytical variations are beyond the scope of this proof-of-concept work. As such, all experiments were performed at room temperature (23° C). Unlike Au sensors, however, for ITO sensors there is no change in signal across all resolvable frequencies (Fig. 5C). This result indicates that surface-bound aminoglycoside aptamers do not undergo binding-induced conformational changes on the ITO surface, a prerequisite for signaling with NBE sensors.

To further confirm the absence of conformation switching behavior in the aminoglycoside ITO sensors, we spiked the cell with single-stranded oligonucleotides complementary to the aptamer. Theoretically, hybridization of the complementary strand should force the surface-attached oligos into an elongated conformation with the attached redox reporter at its maximal distance from the electrode surface. This event should then be detectable by a decrease in signal amplitude on the Lovriç map. Building maps in the absence and presence of 1 *μ*M complementary strand (a surface saturating amount of DNA) though, we still observed no change in signal across all frequencies tested (Fig. 5D). However, coupling reporter-free aptamer strands to the ITO sensors and then hybridizing them to methylene blue-modified complementary strands resulted in large methylene blue currents in voltammetry (Fig. S4), which upon characterization via Lovriç maps matched the signals previously seen in Figs. 5C, D. Overall, these results confirm that DNA-DNA hybridization is occurring on the surface of alkylphosphonic acid monolayers on ITO, but such interaction does not change the spatial positioning of methylene blue relative to the ITO surface in the case of this aptamer.

**Figure 6.**
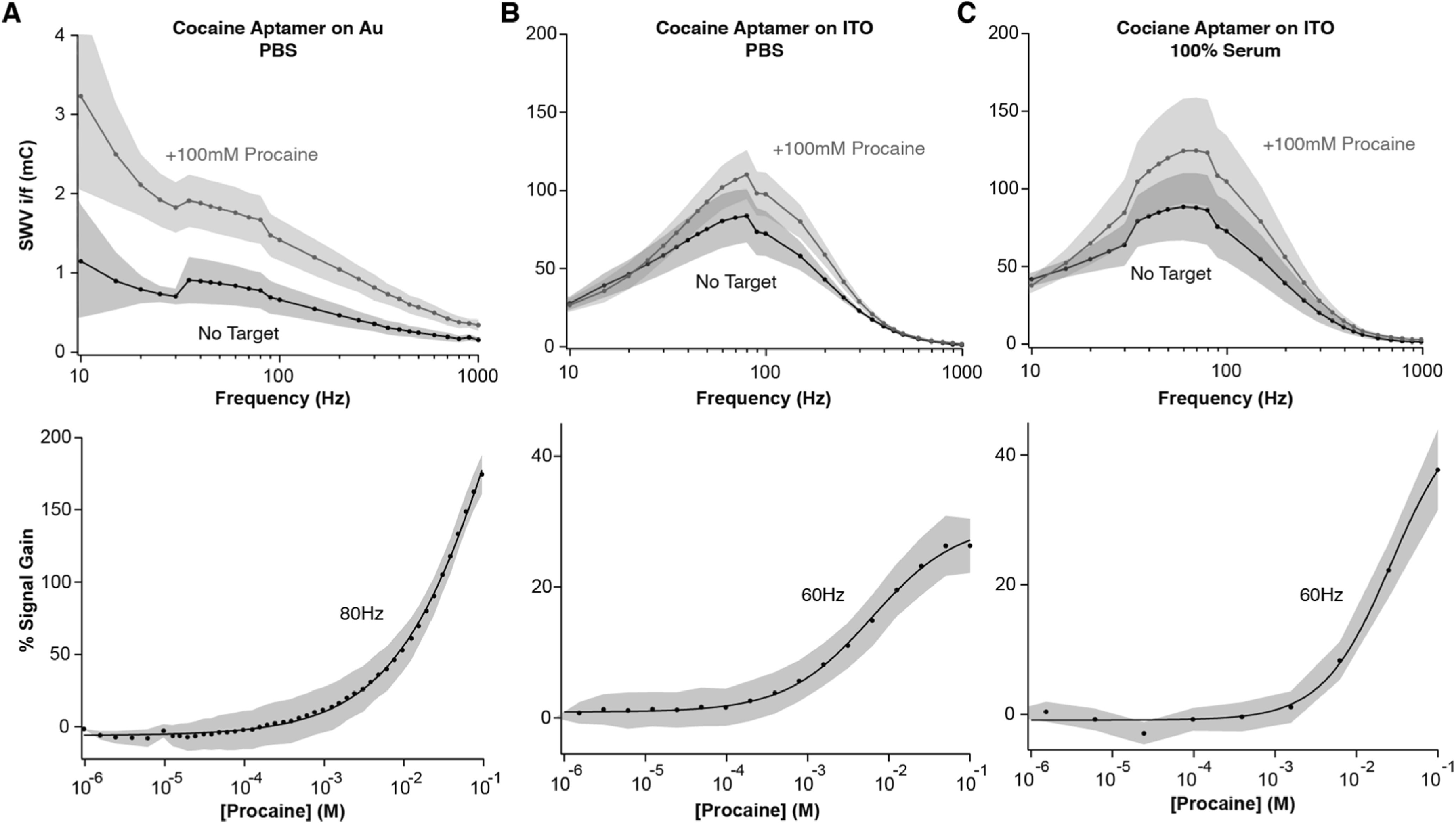
Sensor Signaling with the Cocaine Aptamer. (top) By interrogating cocaine aptamer-functionalized sensors at 27 SWV frequencies, in the absence and presence of a saturating amount (100 mM) of the minor groove-binder procaine, we demonstrate here that (A) Au-supported sensors increase signal upon target binding at all frequencies, (B, C) ITO-supported sensors increase signal in the range of ∼50–300 Hz. (bottom) We then serially interrogate sensors at increasing concentrations of procaine to build calibration curves with (A) ∼200% signal gain and apparent K_D-Au_ = 63.7 ± 9.3 mM for Au sensors, (B) ∼30% signal gain and apparent K_D-ITO_ = 6.2 ± 0.6 mM for ITO sensors in PBS, and (C) ∼48% signal gain and apparent K_D-ITO_ = 27.3 ± 7.5 mM for ITO sensors in 100% human serum. All SWV was performed in the potential window of 0 to −0.4 V vs Ag|AgCl, with an amplitude of 25 mV. Shaded areas represent the standard deviation calculated from at least 4 independent electrodes.

To determine if the above lack of signaling is sequence/construct specific, we functionalized sensors using an aptamer sequence binding procaine (Table I), a small molecule analgesic. Procaine is known to bind the minor groove of DNA^43^ and has been previously reported to have significant binding to the cocaine aptamer,^44^ which we use here. Building Lovriç maps in the absence and presence of 100 mM procaine hydrochloride, we observe a measurable increase in signal upon target binding for both Au- and ITO-supported sensors (Fig. 6, top). For Au sensors, this increase occurs at all frequencies tested (Fig. 6A), while for ITO sensors, it only occurs at frequencies in the range of ∼50–300 Hz (Figs. 6B, 6C). Selecting a frequency with high signal-to-noise measurements (80 Hz for Au, 60 Hz for ITO), we can create binding curves upon increasing concentration of procaine (Fig. 6, bottom). Notably, the signal gain for ITO sensors in this case is ∼4–6 × lower than that for Au sensors. We believe this discrepancy could arise due to the difference in absolute current magnitude between the two surfaces. While the Au sensors have baseline methylene blue currents on the order of hundreds of nanoamps, ITO sensors instead show tens of microamps during interrogation. Because of this large separation in baseline current, small current changes upon procaine binding account for a greater percentage of the signal from Au sensors than the signal from ITO sensors. Alternatively, this may indicate again that the aptamers prefer to remain in a folded conformation on ITO, hence the larger baseline currents. This theory is also supported by the increased affinity displayed by ITO sensors compared to Au (K_D-ITO_: 6.2 ± 0.6 mM; K_D-Au_: 63.7 ± 9.3 mM). If the aptamers are preferentially in their folded conformation on ITO, then a lower concentration of procaine would be needed to bind the minor groove, whereas unfolded aptamers on Au would require higher procaine concentrations to promote aptamer folding and minor groove formation. However, regardless of signal gain magnitude or apparent dissociation constant, both sensors display reproducible signal ON behavior.

Motivated by the procaine NBEs signaling on the ITO surface in PBS, we additionally performed a calibration experiment in 100% undiluted human serum (Fig. 6C). Although there is a 4-fold increase in apparent dissociation constant compared to PBS (K_D-PBS_: 6.2 ± 0.6 mM; K_D-Serum_: 27.3 ± 7.5 mM), the signal gain is higher in serum than PBS, suggesting alkylphosphonic acid monolayers on ITO as promising materials for the creation of biofluid-compatible NBE sensors. We are currently unsure why NBE sensor signaling is sequence/structure dependent on ITO, but this will be the study subject of future work. Nevertheless, our results demonstrate for the first time that NBE signaling is possible with alkylphosphonic acid monolayers on ITO, therefore increasing the scope of substrate materials that can be used for future sensor development efforts.

## Discussion

Although we believe this work is, to our knowledge, the first demonstration of NBE signaling with alkylphosphonic acid monolayers on ITO, we note this is not the first time such monolayers have been reported. The interaction between alkylphosphonates and metal oxides has been known for several decades,^45^ and many studies have investigated their binding modes and methods of monolayer formation.^27^ In 1995, Gardner et al.^26^ brought these monolayers closer to our current application by creating electroactive monolayers on ITO with ferrocene coupled alkylphosphonic acids. Since then, alkylphosphonic acid monolayers have been studied with regards to their binding energy,^46^ electrochemical stability,^47,48^ and deposition solvents.^30^ More recently, Justin Gooding’s group out of the University of New South Wales has brought ITO-bound alkylphosphonic acid monolayers into the realm of biosensor development.^49,50^ In these reports, they use very similar protocols to ours for the deposition, annealing, and functionalization of alkylphosphonic acids. However, unlike the present work, they do not attach oligonucleotides for target binding and signal generation. Instead, they couple a fluorescent dye that does not require surface dynamics for its downstream applications. Therefore, the novelty in our present work lies not in the formation of alkylphosphonic acid monolayers, but in the demonstration of nucleic acid-based sensor signaling using such monolayers.

In this work, we demonstrate successful signaling on ITO sensors for only a singular target thus far. However, we hope to expand this reach in future studies. Due to the relatively small change in capacitance on the ITO surface after monolayer deposition and annealing (Figs. 3, S1), we believe that these monolayers are loosely packed with still a significant amount of exposed oxide surface. This theory could also explain the limited nucleic acid dynamics we see with ITO sensors, as the phosphate backbone of DNA could be interacting with the ITO. One way to potentially increase monolayer packing is to alter the underlying surface itself. The crystallinity of ITO is known to play a role in alkylphosphonic acid monolayer packing, with amorphous ITO supporting higher packing density than polycrystalline ITO.^51^ Since we used commercially available ITO coated glass slides without further alteration before sensor fabrication, we do not know their crystallinity. Precise control of the ITO crystal structure could increase monolayer packing and stability. Another way to alter packing density is by using different solvents during the monolayer deposition step.^30^ We used water in this work for its simplicity and compatibility with biological fluids; however, monolayer deposition from a solvent such as dimethyl sulfoxide or tetrahydrofuran could increase packing density. In future studies, we will investigate how different grades of ITO and different monolayer deposition solvents affect the ability of NBE signaling on ITO.

In addition to alkylphosphonic acids, we also hope to explore more monolayer chemistries for the formation of NBE sensors. Oxide surfaces can be modified with a variety of types of monolayers, including silanes, amines, alkenes, and others. A review article by Pujari et al.^27^ in 2014 describes beautifully the many different attachment chemistries and how they are formed. In the present work, we focused on phosphonates because: (1) alkylphosphonic acid monolayers can be deposited from liquid solutions, providing the benefit of easy fabrication without the need for harsh chemicals or gas deposition chambers; (2) While silane monolayers can also be deposited from liquids, their formation is highly dependent on the organic solvent used and the residual water content of that solvent. Phosphonic acid monolayers, on the other hand, are compatible with aqueous solvents and have stronger binding energy with an oxide surface than silanes; (3) We are interested in eventually deploying our sensors in biological fluids like serum or blood, which are most accurately represented by PBS in terms of buffered solutions. A study by Bhairamadgi et al.^25^ investigated the hydrolytic stability of 22 different oxide surface/ monolayer combinations and found that phosphonic acid monolayers on ITO are more stable than either alkene on ITO or thiol on Au monolayers in PBS. With these three reasons in mind, we chose alkylphosphonic acid monolayers on ITO as our first exploration away from thiols on Au for the formation of NBE sensors. However, we encourage others in the field to join us in attempting additional attachment chemistries to replace thiols on Au, as a platform to translate NBE sensors to novel materials relevant to biomedical and biological applications.

## Conclusions

In this work, we created an example NBE sensor on ITO-coated glass slides. Specifically, by leveraging attachment chemistries between alkylphosphonic acids and an oxide surface, we created covalent monolayers onto which we successfully coupled redox reporter-modified oligonucleotides. Although signaling was not achieved for every sensor architecture attempted, our demonstration of signaling with procaine and the cocaine-binding aptamer presents, to our knowledge, the first NBE senor created with a non-thiol-based monolayer. Examination of the operational stability for this sensor reveals a rapid decay in signal faster than that for analogous Au-supported sensors. However, due to the inherent electrochemical instability of ITO previously discovered by Geiger et al.,^28^ we believe this signal decay is not caused by monolayer desorption, as is the case with traditional thiol on Au NBE sensors.^37^ Despite the instability of ITO, its ease of use and wide-ranging applications provide value for creation of NBE sensors, as ITO-supported sensors could be used for single-use or short term applications. Additionally, ITO’s stability may be altered through better monolayer packing or the use of monolayers other than alkylphosphonic acids. Alternatively, the alkylphosphonic acid monolayers developed in this work could be deployed with longer signaling lifetime on other oxide surfaces. This work provides a starting framework for future exploration of monolayer forming chemistries with the goal of developing NBE sensors on novel materials relevant to biological research and clinical applications.

## Materials and Methtods

### Chemicals and materials

6-mercapto-1-hexanol (MCH, Cat. #451088), 11-mercaptoundecanoic acid (MUA, Cat. #450561), ammonium hydroxide solution (25%, Cat. #17093), hydrogen peroxide solution (50%, Cat. #516813), acetonitrile (Cat. #271004), 11-phosphoundecanoic acid (PUA, Cat. #678031), procaine hydrochloride (Cat. #P9879), and N-(3-dimethylaminopropyl)-N’-ethylcarbodiimide hydrochloride (EDC, Cat. #E7750) were purchased from Sigma-Aldrich (St. Louis, MO). Phosphate buffered saline (PBS, 11.9 mM HPO_3_^2−^; 137 mM NaCl; 2.7 mM KCl; *p*H = 7.4, Cat. #BP399) sulfuric acid (H_2_SO_4_ Cat. #A510), sodium hydroxide (NaOH, Cat. #S318), magnesium chloride hexahydrate (MgCl_2_ Cat. #BP214), and sodium chloride (NaCl, Cat. #S271) were purchased from Fisher Scientific (Waltham, MA). Ethanol (Cat. #111000200) was purchased from Pharmco (Brookfield, CT). New methylene blue zinc chloride double salt (NMB, Cat. #192020100) and guanidine hydrochloride (Cat. #120230010) were purchased from Acros Organics (Fair Lawn, NJ). Tobramycin sulfate (Cat. #T1598) was purchased form Spectrum (Gardena, CA). 6-hydroxy-hexylphosphonic acid (HHPA, Cat. #SIK7301–11) was purchased from Sikemia (Grabels, France). Gender pooled human serum (HUMANSRM-0000351) was purchased from BioIVT.

We prepared all aqueous solutions using deionized water from a Milli-Q^®^ Direct purification system, with a resistivity of 18 MΩ. Basic piranha solution contained a 1:1 volumetric ratio of 25% ammonium hydroxide solution and 50% hydrogen peroxide solution. Coupling buffer contained 250 mM NaCl and 5 mM MgCl_2_. All electrochemical experiments were performed in 1x PBS.

Gold working electrodes (Cat. #002314, diameter: 1.6 mm) were obtained from ALS Inc. (Tokyo, Japan). Platinum wire counter electrodes (Cat. #CH115) and Ag/AgCl reference electrodes (Cat. #CHI111) were purchased from CH Instruments (Austin, TX). For polishing gold electrodes, cloth pads (Cat. #MF-1040) and alumina slurry (Cat. #CF-1050) were purchased from BASi (West Lafayette, IN). Indium tin oxide-coated glass slides (ITO, 15 × 15 × 1.1 mm, 10 Ω resistivity) were purchased from Huanyu (via Amazon). Insulating SECM cells (Cat. #CHI_ICELL) to hold ITO were purchased from CH Instruments (Austin, TX). Copper conductive tape (Cat. #77802) to connect ITO to potentiostat leads was purchased from Electron Microscopy Sciences (Hatfield, PA).

**Table I.** DNA sequences employed in this work.

Target	Sequence	References
Cocaine	5’ - GAC AAG GAA AAT CCT TCA ACG AAG TGG GTC − 3’	52
Aminoglycoside	5’- GGG ACT TGG TTT AGG TAA TGA GTC CC − 3’	39
Aminoglycoside complement (unmodified)	5’ - GGA CTC ATT ACC TAA ACC AAG TCC − 3'	N/A

Oligonucleotides were purchased from IDT. The modifications employed in this work had the following structures:

Amine modification at the 5’ terminus:



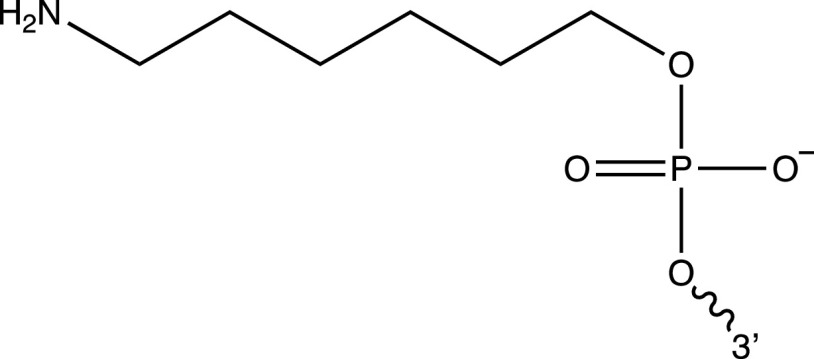



Methylene blue (MB) modification at the 3’ terminus:



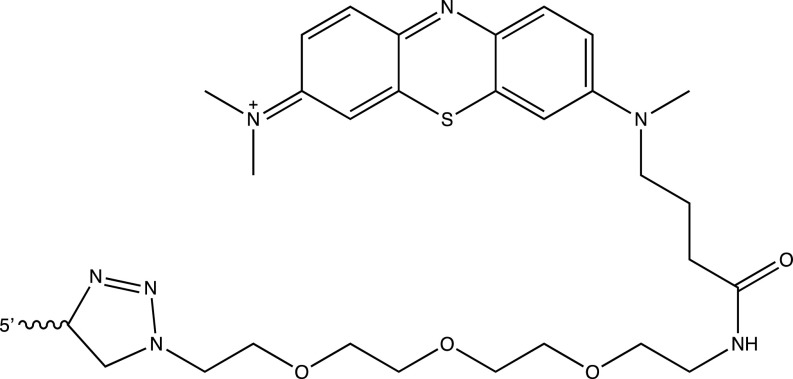



### Gold electrode preparation

Gold electrodes were polished for ∼ 3 min on a cloth pad with alumina slurry. After sonicating (Branson 2800) in ethanol to remove polishing debris, they were then electrochemically cleaned in 0.5 M NaOH and 0.5 M H_2_SO_4_ following a previously reported protocol.^16^ Briefly, (1) in 0.5 M NaOH, we scanned from −0.3 to −1.6 V vs Ag/AgCl, 200 times at a scan rate of 0.5 V s^−1^; (2) in 0.5 M H_2_SO_4_, we scanned from 0 to 1.6 V vs Ag/AgCl, 200 times at a scan rate of 0.5 V/s. Once electrodes showed reproducible voltammograms in sulfuric acid, we rinsed them with water and placed them immediately into a solution of 1 mM MCH, 100 *μ*M MUA, then left them to incubate overnight. To prepare the DNA solutions for attachment to electrode surfaces, we diluted the DNA with coupling buffer ±20 mM EDC to a final concentration of 1 *μ*M, as determined via molecular absorbance measurements employing an Implen Nanophotometer NP80. After incubation with monolayer materials, Au electrodes were placed in the DNA solutions for 2 h. During this time, they were continuously mixed at 1500 rpm using an Eppendorf Thermomixer F1.5. Following DNA coupling to monolayers, non-specifically bound oligos were washed off in a two-step process: (1) mixing at 1500 rpm in acetonitrile for 30 min; (2) mixing at 1500 rpm in 6 M guanidine hydrochloride in 80% ethanol for 30 min Fully fabricated sensors were then placed in a cell with 1x PBS for electrochemical measurements.

### ITO electrode preparation

ITO-coated glass slides were prepared following a multi-step process. (1) Sonication for 10 min in ethanol to remove organic contaminants. (2) Sonication in basic piranha solution for 20 min to oxidize the surface. (3) Incubation in an aqueous solution of 1 mM HHPA, 100 *μ*M PUA overnight to form monolayers. (4) Incubation at 140 °C for 4 h to evaporate interfacial water and promote strong bonding between the monolayer and oxide surface. (5) Incubation in 1 *μ*M solutions of modified oligos overnight. (6) Sonication in acetonitrile for 30 min followed by sonication in 6 M guanidine hydrochloride in 80% ethanol for 30 min to remove non-specifically bound nucleic acids. Fully fabricated sensors were then attached to copper conductive tape and secured into individual electrochemical cells with 1× PBS or 100% human serum for electrochemical measurements. For long-term measurements, cells were also covered with parafilm to prevent solvent evaporation and maintain constant electrolyte concentrations.

### Electrochemical measurements

A CH Instruments Electrochemical Analyzer (CHI 1040 C, Austin, TX) multichannel potentiostat and associated software was used for all CV and SWV measurements. We used a three-electrode cell configuration consisting of gold or ITO working, platinum wire counter, and Ag/AgCl (saturated KCl) reference electrodes. Cyclic voltammetry measurements were recorded at a scan rate of 1 V/s in the potential window 0 to −0.4 V vs Ag/AgCl. Current was sampled every millisecond. SWV measurements were performed from 0 to −0.4 V vs Ag/AgCl with square-wave amplitude of 25 mV, step size of 1 mV, and various frequencies.

### Data analysis

We employed a previously reported, open-source Python script called SACMES^53^ for the processing of our electrochemical measurements. Graphs were created in Igor Pro v8, and dissociation constants were determined via best fit to the Hill equation.
